# Hydrogen Bond Strengthens Acceptor Group: The Curious Case of the C–H···O=C Bond

**DOI:** 10.3390/ijms25168606

**Published:** 2024-08-07

**Authors:** Kingshuk Basu, Esther S. Brielle, Isaiah T. Arkin

**Affiliations:** 1 Department of Biological Chemistry, The Alexander Silberman Institute of Life Sciences, The Hebrew University of Jerusalem, Edmond J. Safra Campus, Jerusalem 9190400, Israel; kingshuk.baso@mail.huji.ac.il; 2 The Alexander Grass Center for Bioengineering, Benin School of Computer Science and Engineering, The Hebrew University of Jerusalem, Edmond J. Safra Campus, Jerusalem 9190400, Israel; esther.brielle@mail.huji.ac.il

**Keywords:** C–H···O hydrogen bond, isotope edited FTIR spectroscopy, molecular dynamic simulation, density functional theory

## Abstract

An H-bond involves the sharing of a hydrogen atom between an electronegative atom to which it is covalently bound (the donor) and another electronegative atom serving as an acceptor. Such bonds represent a critically important geometrical force in biological macromolecules and, as such, have been characterized extensively. H-bond formation invariably leads to a weakening within the acceptor moiety due to the pulling exerted by the donor hydrogen. This phenomenon can be compared to a spring connecting two masses; pulling one mass stretches the spring, similarly affecting the bond between the two masses. Herein, we describe the opposite phenomenon when investigating the energetics of the C–H···O=C bond. This bond underpins the most prevalent protein transmembrane dimerization motif (GxxxG) in which a glycine Cα-H on one helix forms a hydrogen bond with a carbonyl in a nearby helix. We use isotope-edited FT-IR spectroscopy and corroborating computational approaches to demonstrate a surprising strengthening of the acceptor C=O bond upon binding with the glycine Cα-H. We show that electronic factors associated with the Cα-H bond strengthen the C=O oscillator by increasing the *s*-character of the σ-bond, lowering the hyperconjugative disruption of the π-bond. In addition, a reduction of the acceptor C=O bond’s polarity is observed upon the formation of the C–H···O=C bond. Our findings challenge the conventional understanding of H-bond dynamics and provide new insights into the structural stability of inter-helical protein interactions.

## 1. Introduction

Hydrogen bonding is one of the most important forces governing molecular integrity in the physical and biological worlds. It describes the sharing of a hydrogen that is covalently bound to an electronegative Donor atom, with another electronegative Acceptor atom that has a lone pair of electrons: D–H···A.

In the biological world, the acceptor group is often composed of nitrogen or oxygen bound to a carbon. Upon H-bonding, the C–O or C–N bonds are weakened due to the pulling exerted on the electronegative atom by the hydrogen. In the current study, we identified the opposite effect, wherein the acceptor of an H-bond is surprisingly strengthened upon H-bonding. Consequently, by looking into the governing factors of H-bond formation, we aimed to examine the roots of this unexpected and intriguing observation.

Theoretical and experimental findings describe the formation of hydrogen bonds due to several energy components: electrostatic attraction, electron delocalization, charge transfer, dispersive interactions, cooperative effects, steric effects, and secondary interactions [1].

H-bonds in proteins exhibit variations in these energy components, tailored to fulfill the specific physiological role of the molecule [2]. The nature of H-bonds in proteins is further influenced by the molecular makeup of the donor and acceptor, the protein backbone, and the side chain [3,4].

One can classify protein and peptide H-bonds as canonical or noncanonical. Canonical or conventional H-bonds are formed by regular and predictable H-bonding interactions, like the *i* to i+4 H-bonds formed between the C=O and N–H groups in α-helices, respectively [5]. Noncanonical H-bonds exhibit a significantly larger variation of donor and acceptor groups and can include a multiplicity of partners. For example, an over-coordinated system entails several donors bonded to a single acceptor, while multifurcation involves a single donor with multiple acceptors. Experimental and computational analyses have shown that some of these noncanonical bonds are highly important for protein structure [6,7].

An important class of noncanonical H-bonds in proteins are those formed between a Cα hydrogen and a carbonyl oxygen [8,9,10,11,12,13,14,15]. X-ray and neutron crystallography have confirmed the identity of Cα-H···O=C hydrogen bonds within the protein environment in the presence of a solvent, other proteins, and ligands [16,17], suggesting an essential role in catalytic activity [18]. These important hydrogen bonding schemes necessitate detailed analyses to provide a quantitative understanding of their role in macromolecules.

The Cα-H···O=C hydrogen bond in a transmembrane helical dimerization interface was first identified in glycophorin A [19]. The interface has a GxxxG motif, one of the most prevalent oligomerization factors in transmembrane helices [20,21]. In the GxxxG motif of glycophorin A, the Cα-Hs of glycines 79 and 83 form H-bonds with the carbonyls of isoleucine 76 and valine 80 in the opposing helix, respectively. Such bonds form the basis of the GxxxG dimerization motif [13].

Previous attempts to measure the energetics of the Cα-H···O=C H-bonds resulted in varying estimates. Using a mutagenesis cycle analysis, Bowie and coworkers found that such bonds are not stabilizing [22], while an empirical FTIR-based approach indicated a bond enthalpy of 0.88 kcal/mol [23].

Considering the prevalence of the Cα-H···O=C interaction and its contested contribution to protein stability, we decided to examine the energetics of Cα-H···O=C hydrogen bonds using a combination of experimental and computational methods. Experimentally, we employed vibrational spectroscopy, an exquisitely sensitive tool to measure H-bond strength. Computationally, we use molecular dynamic simulations and DFT calculations that can yield detailed energetics and vibrational shifts that can be verified with experiments. Specifically, we targeted the frequency change of the C=O acceptor upon H-bond formation, made possible by isotopic labeling to resolve the specific carbonyl group [24,25]. This combined approach enabled us to both measure the strength of this interaction and explain the source of its curious spectroscopic behavior.

## 2. Results and Discussion

**Figure 1:**
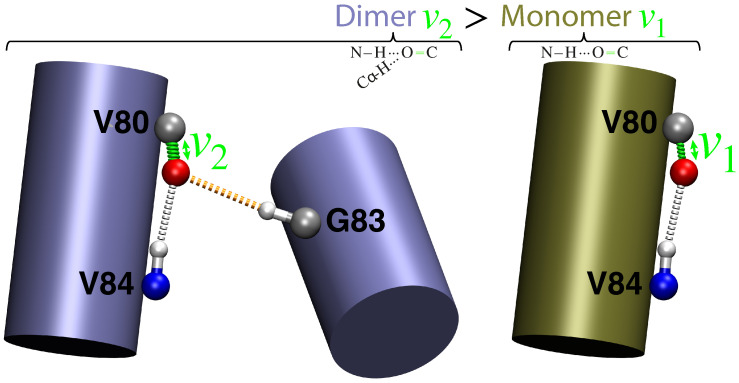
Graphical representation of the homo dimer (**left**) and monomer (**right**) species showing the presence or absence of the noncanonical C–H···O bond, respectively. The impact on the C=O bond and its corresponding vibration frequency is shown in green.

Glycophorin A is the first membrane protein to have its sequence determined [26] and is the archetypical GxxxG containing transmembrane domain dimer [13,19,27]. The GxxxG motif enables close positing of the two helices with minimal steric hindrance due to the small size of the glycine hydrogen side-chain that H-bonds to a carbonyl residue in the other helix. Figure 1 depicts the bond between the Cα-H of Gly83 and the carbonyl acceptor of Val80. Note that the acceptor is also involved in a canonical H-bond with the backbone amide donor hydrogen Val84. Hence, Val80’s carbonyl group participates in an over-coordinated H-bonding interaction.

**Figure 2:**
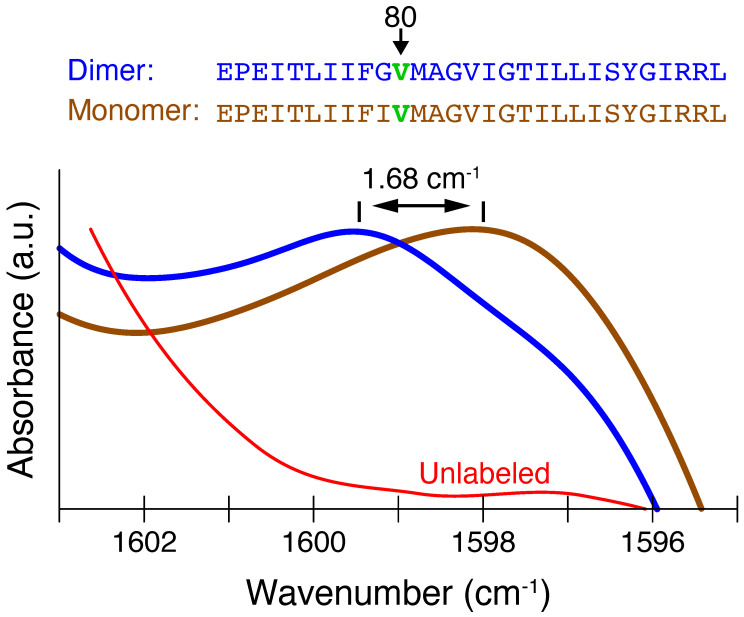
Amide I stretch of Val80 ^13^C=^18^O isotopically labeled dimeric (with C–H···O H-bond) and monomeric (without H-bond) species, depicted in blue and brown, respectively. The spectrum of a peptide without isotopic labels is shown in red. Top: sequences of the dimeric (blue) and monomeric (brown) peptides used in the study, indicating the position of the^13^C=^18^O label at Val80 in green.

We spectroscopically isolated the Cα-H···O=C bond in Glycophorin A by editing Val80’s carbonyl group with ^13^C and ^18^O. Due to the fact that the amide I mode is composed mainly of the C=O stretch [28], such labeling baseline resolves the isotope-edited mode from the unlabelled amide groups by shifting it more than 60 ${\mathrm{cm}}^{-1}$, enabling detailed site-specific analysis of the labeled peak separately from the unlabeled peak [25]. Accordingly, as shown in Figure 2, the ^13^C=^18^O amide I mode of Val80 within glycophorin A is shifted to 1599.4 ${\mathrm{cm}}^{-1}$, which is 62 ${\mathrm{cm}}^{-1}$ from the main amide-I envelope of the protein. The entire spectra of both peptides, in which all other vibrational modes arising from the lipid and protein can be observed, are presented in Figure S1.

The peptide encompasses the transmembrane domain of wild-type glycophorin A, a strongly dimeric species [27,29,30] in which the Cα-H···O=C hydrogen bond exists [13,19]. Therefore, to determine the impact of the Gly83–Val80 inter-helical H-bond, we measured the spectrum obtained from a peptide containing the Gly79Leu monomerizing mutation that separates the helices, and so the bond does not exist [27].

In the monomeric protein that does not contain the noncanonical H-bond, the isotope-labeled amide I mode resonates at 1596.8 ${\mathrm{cm}}^{-1}$, which is *lower* than the H-bonded dimeric species by 1.7 ${\mathrm{cm}}^{-1}$ (Figure 2). These results appear surprising since H-bond formation is expected to reduce the vibrational energy of the acceptor carbonyl and thus shift the isotope-edited carbonyl peak to lower frequencies when it is involved in a hydrogen bond.

We note that previous studies report a blue shifting of an H-bond donor. For example, using computational tools, Schlegel and colleagues have elucidated the electrostatic origin of this shift [31]. However, to the best of our knowledge, we are reporting for the first time the blue shift of the stretching frequency of an H-bond *acceptor* C=O in proteins.

**Figure 3:**
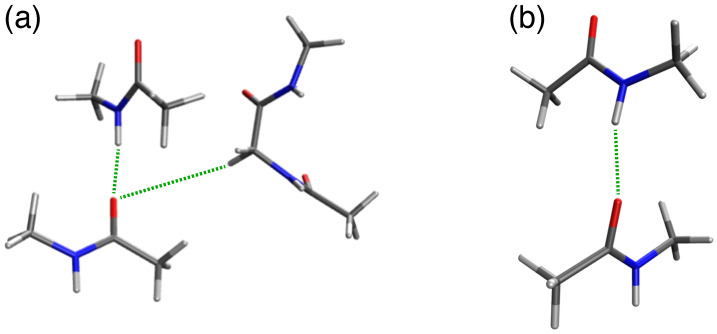
Energy-optimized representative model assembly of (**a**) two N-methylacetamides and acetylglycinemethylamide, mimicking the C–H···O interaction of the glycophorin A dimer after DFT energy minimization. (**b**) Two N-methylacetamides mimicking the canonical C=O···H–N interaction of the glycophorin A monomer with the G79I mutation (the C–H···O inter-helical hydrogen bond is absent here), after DFT energy minimization. H-bonds are shown as green dotted lines.

To understand the root of the surprising frequency shift, we employed DFT-based computational tools that detail the characteristics of such H-bonds [32]. The size of a transmembrane helix system is beyond the current capabilities of detailed quantum calculations. Therefore, two mimetic systems that capture the specific H-bonding interactions were analyzed and compared (Figure 3 and Figure S4):

*Monomer (single H-bond)*: The canonical α-helical H-bond between the carbonyl of valine 80 and the amide H of glycine 84 was mimicked by two N-methylacetamides.
*Dimer (two H-bonds)*: The inter-helical H-bonding system contained the canonical H-bond described above, with an additional noncanonical inter-helical hydrogen bond. The Cα-H of glycine 83 from the opposing helix is bonded to the same carbonyl of valine 80 that is also involved in the canonical hydrogen bond. This system was mimicked by two N-methylacetamides forming the canonical H-bond to which an acetylglycinemethylamide is hydrogen bonded.

To ensure that the calculations replicate the experimental system accurately, we sought to superimpose the coordinates of the atoms on the corresponding groups of the transmembrane protein. However, only the structure of the wild-type, dimeric glycophorin A, has been solved experimentally [19,33]. Moreover, the structures were elucidated in micelles or bicelles but not in a lipid bilayer, the native environment of the protein and the one in which the FTIR spectra were obtained. Therefore, we used molecular dynamics (MD) simulations in hydrated 1,2-dimyristoyl-sn-glycero-3-phosphocholine bilayers to determine the atom positions in the wild-type, dimeric glycophorin A and a monomeric glycophorin A peptide that contains the G79I mutations. In both instances, the experimentally determined structure in bicelles was used as a starting point [33]. The results of the MD simulation can be seen in Supporting Information, Figure S2, which depicts structures of both monomer and dimer species, and Figure S3, which shows the root mean square deviation for each species.

We extended the utility of the MD simulations beyond creating mimetic systems for density functional theory (DFT) calculations, applying them also to geometric analyses of bond parameters. The geometry of a bond, and in particular its distance and angle, influences its energetics and, consequently, the vibrational frequency. Previous research has highlighted the variability of H-bond distances along helical peptides, discussing a trend of bond shortening at helix midpoints due to the cooperative effect of H-bonding along the length of the helix [34]. Similarly, Tan et al. have shown how the strength of an H-bond is dependent on the donor-acceptor angle, introducing the concept of an ‘antecedent angle’, which varies across protein secondary structures [35].

We calculated hydrogen bond distances and angles within our simulated systems. Specifically, we found the average O···N distance between Val80’s C=O and Val84’s N–H along the same helix to be 2.88±0.06 Å in the dimeric species of glycophorin A, compared to 2.97±0.16 Å in the monomeric variant. On the other hand, the C–O···N bond angle was 163.35±2.7° in the dimer and 160.81±9.4° in the monomer.

Hydrogen bonding interactions between donor and acceptor moieties can be both dipolar and electronic. An electronic interaction is possible when the donor N-H anti-bonding orbital overlaps the C=O oxygen nonbonding orbital. Electronic interactions are optimal when the C–O···N angle is near 120° [36], facilitating maximal overlap of these orbitals. But in protein systems, there are typically geometric constraints that restrict such bond angles to around 155° [35,37].

In the dimeric model, with the additional inter-helical H-bond, a 3° wider C–O···N bond angle is observed relative to the monomeric model. This finding points to a reduced C=O to N–H canonical hydrogen bond strength. On the other hand, the canonical H-bond distance upon dimer formation was shortened by 0.09 Å, potentially increasing orbital overlap and thus increasing the canonical C=O···H–N interaction strength. Given the competing influences observed, MD simulations alone do not provide a definitive conclusion on the dominant effect on whether the canonical hydrogen bonding strength should increase or decrease in the dimer system versus the monomer system. Such ambiguity is perhaps not unexpected due to the simplicity of the point charge model of the MD force field). Therefore, we proceeded to conduct quantum mechanical calculations for each system in which the C=O oscillator strength and other parameters can be estimated with greater accuracy, and the hydrogen bond strength is directly inferred.

We calculated the frequencies of the amide I stretching modes (ν_C=0_) of both the monomeric and dimeric systems. To make our calculation more accurate and statistically significant, we extracted coordinates from several frames from the MD simulation trajectory based on minimal RMSD values and subjected them to DFT calculations. From each frame of dimeric and monomeric species, coordinates of Val80 and Val84 were extracted from one helix and only for the dimeric species, coordinates of Gly83 were extracted from the neighboring helix. Using these coordinates, N-methylacetamide molecules were constructed representing Val80 and Val84. Gly83 (for the dimer) was kept intact with the capping of CH_3_ groups at terminal –NH– and –CONH– groups. Figure 3 shows the energy-optimized constructs for the (a) dimeric and (c) dimeric species.

The average Val80 ν_C=0_ value for the dimeric glycophorin A is 1656.1 ± 2.4 ${\mathrm{cm}}^{-1}$, whereas for the monomeric species (with the G79I mutation) the frequency is 1652.4 ± 2.3 ${\mathrm{cm}}^{-1}$. Notably, there is a 3.7 ${\mathrm{cm}}^{-1}$ increase in the C=O stretching upon dimer formation, which corresponds with the FTIR experimental ν_C=0_ shift upon dimer formation. Hence, the surprising C=O bond strengthening is obtained both experimentally and computationally. The observed shift is independent of isotopic substitution. The ^12^C=^16^O variant of the construct produced a shift of 4.1 ${\mathrm{cm}}^{-1}$ comparable to the ^13^C=^18^O analog that yielded an average shift of 4.2 ${\mathrm{cm}}^{-1}$, arising from the stretching frequencies 1719.6±2.3 ${\mathrm{cm}}^{-1}$ for dimer and 1715.4±2.6 ${\mathrm{cm}}^{-1}$ for monomer.

In a typical H-bond, the acceptor (C=O in this instance) transfers electrons to the donor anti-bonding orbital, reducing the double bond character of the C=O reflected as a decrease in ν_C=0_. The H-bond of a C=O···H–D undergoes a hyperconjugative C^+^–O–H···D^−^ type interaction (analogous to charge transfer) that, in turn, lowers the double bond character of C=O [38].

To explore the electronic basis of the observed acceptor vibrational mode strengthening of the dimer system (upon over-coordination with the additional inter-helical hydrogen bond), we employed Natural Bonding Orbital (NBO) calculations to analyze the estimated electron distributions and atomic orbitals in a localized way. Local hybrid orbitals can be extracted using the NBO approach, which can correlate with classical qualitative bonding theories [39]. Alabugin et al. have shown how imperfect H-bonds can be analyzed by the NBO approach by showing the hyperconjugation effects [38].

According to valence bond theory, a C=O bond should be formed by the overlap of two *sp*^2^-orbitals from the C and O atoms to form a σ-bond, and two *p*-orbitals from the C and O atoms to form a π-bond. Yet, in reality, this picture changes depending on the electronegativity of each participating atom, as explained by Bent’s Rule [40]. However, for an ideal situation of a double bond, a σ-bond (like in the Val84 amide C=O) should project an *sp*^2^ hybrid orbital to the other atom (O in this instance) present in the covalent bond, making the bond $33\frac{1}{3}$% *s* and $66\frac{2}{3}$% *p*. A deviation from these percentages will weaken the bond. In contrast, the π-bond should be formed by two unhybridized 100% *p*-orbitals with no *s*-character.

Upon taking the hyperconjugative effect into account, the C=O bond should lose some of its double bond character due to the presence of a C^+^–O–H species and the localized orbitals will be far from an ideal *sp*^2^ overlap that we would expect in the σ bond.

**Figure 4:**
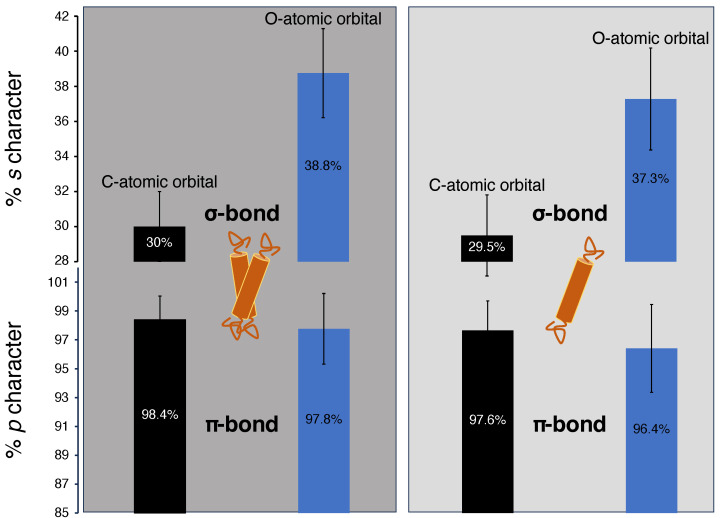
Graphical representation of calculated orbital *s*/*p*-character. The left panel shows calculations on dimeric glycophorin A, and the right panel shows monomeric G79I mutated glycophorin A.

We ran NBO calculations on the energy-optimized DFT structures collected from MD-simulation trajectories and extracted the values of the *s*-character of the C and O atomic orbitals of the C=O bonding interactions. The results of the calculations, shown in Figure 4, indicate that both the monomeric and dimeric species experience a deviation in the *s*-character of the σ and π-bonds from the ideal, $66\frac{2}{3}$% and 100% *s* and *p*-characters, respectively.

The dimeric form has a higher *s*-character in the σ-bond and a higher *p*-character in the π-bond compared to the monomeric form. The average *s*-character of the atomic orbitals contributing to the σ bond of C=O are shown in Figure 4, upper panels. It can be seen that the C and O hybrid orbitals possess a slight but definite higher *s*-character than the corresponding monomeric species. The monomer has a 29.5% and 37.3% *s*-character in its C and O hybrid orbitals, respectively. On the other hand, the dimeric species has a 30% and 38.8% of *s*-character in the respective orbitals. Therefore, upon dimerization and formation of the additional C–H···O bond, the C–O σ bond becomes stronger due to its higher *s*-character, which can be a major contributing factor for the increased ν_C=0_ stretching frequency. This kind of *s*-character enhancement has been previously encountered and has been accounted as the main factor for bond strengthening of the H-donor [38].

On the other hand, the π-bonding C and O atomic orbitals in the dimer are 98.4% and 97.8% of *p*, respectively, whereas for the monomer they are 97.6% and 96.4% of *p*, respectively (Figure 4). So the C=O_dimer_ has more *p*-character in the sidewise overlapping orbitals than in the C=O_monomer_, suggesting reduced hyperconjugation, which should theoretically enhance the oscillator strength of the C=O bond, supporting the observed frequency increase [38]. Finally, we note that indistinguishable results were obtained upon using a dispersion correction (D3_BJ) [41] method with a larger basis set (cc-pVTZ) [42].

These computational results suggest that the C=O bond strength increases upon dimerization, and the formation of the second H-bonding C–H group, due to the increased *s*-character of the σ bond and a simultaneous increase in the *p*-character of the π bond, which reduces hyperconjugation. Altogether, this leaves the C=O bond more double bonded in nature as observed in the later dispersion-corrected calculation.

We followed by analyzing how H-bonding impacts the charges of Val80’s carbonyl group. When the C=O···H–C interaction is absent, either in the monomeric system or in a separated dimeric assembly (Figure 3 and Figure S5), a polar distribution is obtained: C^+0.45^=O^−0.71^ or C^+0.60^=O^−0.65^, respectively. In contrast, when the C=O···H–C is present (dimeric assembly), a markedly different charge distribution is obtained: C^−0.21^=O^+0.09^.

Lowering the C=O bond’s polarity should increase its covalent character. This factor, together with reduced hyperconjugation, potentially contributes to a stronger C=O bond.

It is worth mentioning that less hyperconjugation and a reduction in bond polarity do not imply that the C–H···O H-bond is energetically unfavorable. H-bonding involves both electronic and polar counterparts. Electronic interactions involve the transfer of electrons and the formation of charge-transfer species, whereas dipolar interactions are solely electrostatic in nature. An analysis of the energy terms associated with the canonical and noncanonical H-bonds in both monomer and dimer species reveals this complexity.

There is a small destabilization in the canonical H-bond of the dimer compared to the monomer, with the dimer having a canonical H bond energy value of 5.36±0.12 kcal/mol compared to that of the monomer at 5.71±0.18 kcal/mol. Hence, both bonds impact one another and are consequently not orthogonal in nature [43]. On the other hand, the noncanonical C–H···O H-bond in the dimer has a bond energy of 0.65±0.10 kcal/mol, which indicates an overall stabilization of the dimer species due to the incorporation of two H-bonds. In other words, the additional H-bond from the glycine Cα-H strengthens the interaction between the two helices. Finally, the value of this additional noncanonical C–H···O H-bond is similar to previous measurements [23].

In short, the mechanism of increase in stretching frequency can be summarized as a combination of geometrical and electronic factors as follows: *(i)* The incoming Cα-H group changes the geometry of the canonical C=O···H–N geometry to a finite extent. Though the change in canonical bond angle and bond distances are inconclusive at first sight, the hydrogen bond energy has been reduced to a small but finite extent. *(ii)* the incoming Cα-H has a lower propensity to participate in hyperconjugative interaction with C=O, as evident from the increase in % *s* and *p* of the bond and a change in charge distribution pattern. These two factors reinforce each other to increase the C=O bond’s double bond character and stretching frequency.

## 3. Materials and Methods

### 3.1. Sample Preparation

#### 3.1.1. Isotopic Label Synthesis

Isotopic labeling was conducted as described previously [44]. Briefly, 4.52 mmol of 3,5-dimethylpyridine hydrobromide (Sigma-Aldrich; Rehovot, Israel) in 2 mL of anhydrous N,N-dimethylformamide (Sigma-Aldrich) was mixed with 2.24 mmol of N-(3-(dimethylamino)propyl)-N’-ethylcaerbodiimide hydrochloride (EDC·HCl) (Sigma-Aldrich) and with 11.3 mmol of H_2_^18^O (Sigma-Aldrich) under N_2_ atmosphere. 225 µmol of 1-^13^C–N –FMOC L-valine (Cambridge Isotope Laboratories, Inc; Andover, MA, USA), dissolved in 3 mL of anhydrous N,N-dimethylformamide, was added into the above-stated mixture. Subsequently, the reaction vessel was kept at room temperature and stirred overnight. After allowing the reaction to proceed overnight, another portion of 2.24 mmol EDC·HCl was added in the morning, followed by a third addition of 2.24 mmol EDC·HCl after approximately seven hours, and the reaction was allowed to continue overnight. Afterward, approximately 30 mL of ethyl acetate (Gadot-group; Netanya, Israel) was added. The mixture was transferred to a separatory funnel and washed three times with 0.1 M citric acid, and then once with brine. Ethyl acetate was then added to the combined citric acid and brine portions and separated. The combined portions of ethyl acetate, containing the labeled amino acid, were dried over anhydrous sodium sulfate (Da-sit Group; Milan, Italy) and filtered to remove any extra water. The ethyl acetate was removed by rotary evaporation, creating an azeotrope with methylene chloride (Gadot-group).

#### 3.1.2. Peptide Synthesis and Purification

The labeled valine (see above), was incorporated into two different peptides corresponding to the transmembrane domain of glycophorin A [29]. Two peptides included the native sequence with Valines 80 labeled as well as an G79I [27] mutant with Valines 80 labeled in boldface (sequence starts at residue 70):

EPEITLIIFG **V** MAGVIGTILLISYGIRRL

EPEITLIIFI **V** MAGVIGTILLISYGIRRL

Both of the peptides were synthesized separately with N-(9-fluorenylmethoxycarbonyl) solid-phase chemistry. Each peptide sample was purified with high-performance liquid chromatography on a 20 mL Jupiter 300 Å C4 5 µm high-performance liquid chromatography column (Phenomenex; Torrance, CA, USA). The column was pre-equilibrated with 80:8:12 (by volume) water/acetonitrile/isopropanol, where all solvents contained 0.1% trifluoroacetic acid (TFA) (Merck; Darmstadt, Germany). Approximately 2 mg of protein sample was dissolved in 2 mL of TFA and injected into the column. The solvent gradient was linearly altered with the VWR Hitachi Chromaster 5160 Pump (Tokyo, Japan) to remove all water composition while retaining the acetonitrile/isopropanol ratio at 40%:60% with 0.1% TFA. Peptide elution was monitored at 280 nm using the VWR Hitachi Chromaster 5410 UV detector.

#### 3.1.3. Peptide Reconstitution

All experimental measurements were performed on peptides in lipid vesicles. We used organic solvent co-solubilization in order to reconstitute each peptide in a membrane bilayer. Approximately 1 mg of purified protein and 10 mg of 1,2-dimyristoyl-sn-glycero-3-phosphocholine (Avanti Polar Lipids; Alabaster, AL, USA) were dissolved in 1 mL of 1,1,1,3,3,3-hexafluoro-2-propanol (HFIP) (Merck, Darmstadt, Germany). The mixture was rotary evaporated at 37 °C until all the HFIP evaporated. One milliliter of water was added, and the mixture was rotated at 37 °C to spontaneously form vesicles. The sample was then sonicated to ensure uniformly sized vesicles and no aggregation.

### 3.2. FTIR Spectroscopy

For each of the two samples of peptides in a membrane vesicle, separate FTIR spectra were collected. First, 200 µL of sample was deposited on a germanium trapezoid ATR plate (50×2×20 mm) with a 45° face angle (Wilmad; Vineland, NJ, USA). Following removal of bulk solvent, the crystal was incorporated into a 25-reflection variable angle ATR unit (Specac; Orpington, UK), which reflects the incoming FTIR beam 25 times before its exit from the crystal. The ATR unit was incorporated within a Nicolet iS10 FTIR spectrometer, with a mercury cadmium telluride detector (Thermo Scientific; Waltham, MA, USA), cooled with liquid nitrogen. The FTIR spectrometer was purged with water- and CO_2_-depleted air, and spectra were collected at room temperature.

For each sample, 1000 scans were sampled and averaged at a data spacing of 0.241 ${\mathrm{cm}}^{-1}$ with two levels of zero filling, N-B strong apodization, and Mertz phase correction. For each of the two samples of peptides in a membrane vesicle, separate FTIR spectra were collected at room temperature. Prior to deposition of the sample on the germanium ATR plate, background spectra were collected with an empty germanium ATR plate and used to subtract background IR absorption.

### 3.3. Computational Details

#### 3.3.1. Molecular Dynamic Simulations

The PDB structure of the dimeric glycophorin A dimer (2KPF) was downloaded from RCSB PDB. The monomer was created by stripping out chain B from the dimeric structure and mutating G79 to I79 using Swiss-PDBeditor [45]. Both of the structures were then fitted into a pre-equilibrated 1,2-dimyristoyl-sn-glycero-3-phosphocholine bilayer obtained from supporting data of the study by Poger & Mark [46] to satisfy and mimic the experimental conditions. The fitting was performed in such a way that the helical bundle(s) remain perpendicular to the bilayer plane. The bilayer contains 228 lipid molecules with 4166 water molecules. After initial structural alignment, lipid molecules that coalesced with protein coordinates were removed using InflateGro methodology developed by Tieleman and co-workers [47]. In this method an automatic running algorithm deletes molecules within the 2 Å range. Position restraints (10,000 kcal ${\mathrm{mol}}^{-1}$ ${Å}^{-2}$) on heavy atoms of protein molecules were imposed to make sure that the protein molecules do not change positions during the energy minimization process. Energy minimization was performed with the steepest descent minimization algorithm with a tolerance of 500 kJ ${\mathrm{mol}}^{-1}$ ${\mathrm{nm}}^{-1}$.

Molecular dynamic (MD) simulations of the monomeric and dimeric protein were performed for 200 ns using Gromacs version 2022.3 [48,49,50,51,52] using an extended version of GROMOS96 53A6 force field [53]. The length and angles of H-atoms were restrained with the LINCS algorithm allowing an integration time step of 2 fs [54]. Atomic coordinates were saved every 1000 ps. Reference temperatures were kept at 323 K and solvent, lipids, and proteins were separately coupled to a Nosé-Hoover temperature bath [54,55] with a coupling constant value τ = 0.5 ps. Pressure coupling was performed with a Parrinello–Rahman barostat with τ = 2 ps [56,57]. A 1.2 nm distance was set as cut-off for van der Waals interactions. At every 10 fs the neighbor list was updated. 4th order Particle Mesh Ewald (PME) long-range electrostatics was used to calculate electrostatic parameters [58].

The final simulation box had 225 lipid molecules. Hydration with FLEXSPC model [59] water molecules and addition of Na^+^ and Cl^−^ as counter ions led to a number of atoms of 25598 and 37365 for the monomer and dimer protein systems, respectively.

#### 3.3.2. Quantum Mechanical Calculations

The protein H-bonding system was modeled with smaller molecular mimics. Our focus is the Val80 residue, which is the proton acceptor in the C–H···O bond while simultaneously participating in a canonical H-bond with its H-bond-donating Val84 counterpart from the same helix. We represent this canonical H-bond between Val80 and Val84 with two N-methylacetamide molecules. The noncanonical H-bond-donating glycine moiety from the neighboring helix was modeled with an acetylglycinemethylamide. Schematic representations of the chemical formulae are shown in Figure S4.

Besides atoms in the capping CH_3_ groups, all other atoms of the three molecules are derived from the original protein. Coordinates were chosen from the structure of glycophorin A and its monomer from different frames of MD simulations trajectory based on minimal RMSD values. The C atoms of the capping CH_3_ groups were positioned where the C and Cα atoms were located in the PDB structures. Atom coordinates were selected from chains A and B of the structure, and the terminal C atoms of the N-methylacetamides and glycine were fixed during minimization. The α-carbon of the glycine-mimicking molecule was similarly fixed.

While determining H-bonding energy, we required breaking the C–H···O H-bond. Breaking was accomplished by moving the glycine residue 30 Å apart from the original position, shown schematically in Figure S5. The ^13^C=^18^O system of Val80 was achieved by using ^13^C and ^18^O isotopes in the carbonyl group of the Val80-mimicking N-methylacetamide residue.

The computation was performed using a Q-chem [60] set up at the B3LYP level of theory [61,62,63,64,65] using the “aug-cc-pvdz” basis set [66]. Such large basis sets reduce the chance of basis set superposition error. Moreover, the basis set superposition error was corrected using the DFT-C method available in the Q-chem package [67]. Furthermore, no difference was obtained upon repeating the computational analyses at the same level of theory (B3LYP), using a dispersion correction (D3_BJ) [41] method and with a larger basis set (cc-pVTZ) [42] to further minimize the effects of basis set superposition error.

The dielectric constant was fixed at 4 to mimic the membrane environment. Section S1 of Supporting Information shows the detailed methodology of the computational procedure. The structures of the model molecular assembly after energy minimization can be seen in Figure 3 and are listed in Tables S1–S14.

Q-chem package provides a natural bonding orbital (NBO) calculation tool for measuring natural bonding orbital contributions involved in the chemical bondings. NBO outputs were extracted from the Q-chem output as percent orbital contributions. NBO output also involves natural population analysis (NPA) for each construct, which was also extracted.

## 4. Conclusions

Taken together, we examined the Cα-H···O=C H-bond at the atomic level, using a synergistic combination of experiment and computation focusing on the carbonyl acceptor group. To our surprise, both lines of investigation show a blue shift of the acceptor stretching frequency indicative of a strengthening of the C=O bond, despite previous studies that have shown that proton acceptor groups often have reduced internal bonds upon increased hydrogen bonding. Molecular dynamic simulations followed by DFT calculations reveal that the C-H group cannot undergo effective hyperconjugation thereby increasing the C=O oscillator strength.

## Data Availability

Data is contained within the article or Supplementary Material.

## Supplementary Materials

The following supporting information can be downloaded at: https://www.mdpi.com/article/10.3390/ijms25168606/s1.

## Abbreviations

The following abbreviations are used in this manuscript:

FTIR	Fourier transform infrared
DFT	density functional theory
MD	molecular dynamics
