# Supplementary material for: Hydrogen Bond Strengthens Acceptor Group: The Curious Case of the C–H···O=C Bond
Source: Int J Mol Sci. 2024 Aug 7;25(16):8606. doi: 10.3390/ijms25168606 (PMC11354782; doi:10.3390/ijms25168606)
Supplement: Supplementary file 1 [file ijms-25-08606-s001.zip › ijms-3113423-supplementary.pdf]

Supplementary Materials: Hydrogen Bond Strengthens Acceptor Group: The Curious Case of The C−H···O=C Bond

Kingshuk Basu[1,‡], Esther S. Brielle[2,‡,†] and Isaiah T. Arkin [1,*]

Section S1. Method and coordinates used for quantum mechanical calculations:

The input file for the quantum mechanical calculations is provided below:

```
$molecule
Coordinates from different frames
$end
$rem
JOBTYPE = OPT
exchange = B3LYP
BASIS = aug-cc-pvdz
mem_static = 6000
mem_total = 16000
geom_opt_max_cycles = 500
max_scf_cycles = 500
solvent_dielectric = 4
$end
```

The output from the above process was then fed to the following section for Frequency calculations

```
$opt
fixed
1 xyz
2 xyz
4 xyz
5 xyz
6 xyz
7 xyz
8 xyz
9 xyz
10 xyz
12 xyz
13 xyz
14 xyz
15 xyz
16 xyz
17 xyz
18 xyz
19 xyz
20 xyz
21 xyz
22 xyz
23 xyz
endfixed
$end
```

```
@@@

$molecule
READ
$end

$isotopes
1 0
2
2 13.00335
3 17.99916
$end

$rem
jobtype freq
exchange B3LYP
BASIS aug-cc-pvdz
scf_guess READ
solvent_dielectric 4
max_scf_cycles 500
ISOTOPES 1
$end
```

The following commands were used for NBO calculations of the optimised structures:

```
$rem
BASIS = aug-cc-pVDZ
GUI = 2
METHOD = B3LYP
NBO = 2
$end
```

Section S1.1. Coordinates for dimeric species:

Table S1. Frame 1

0 1			
C	-2.1560194	-3.1366684	0.6863450
C	-2.9741717	-2.0800987	-0.0647124
O	-2.4202696	-1.0922180	-0.5616273
N	-4.2906484	-2.2455326	-0.0527623
H	-4.6540927	-3.0522103	0.4302265
C	-5.2789756	-1.2234018	-0.4129816
C	-1.2726328	1.5754521	1.8912177
C	-1.2881271	2.6351288	0.7878387
O	-1.2859753	3.8499380	0.9879310
N	-1.7443679	2.1329905	-0.3587567
H	-1.8118593	1.1243582	-0.4445931
C	-2.0219171	2.8769947	-1.5958670
C	4.0674315	-3.0216528	0.7869918
C	3.3019077	-2.1369373	-0.2021755
O	3.8151635	-1.6513206	-1.2123957
N	2.2079742	-1.5496794	0.2598174
H	1.7129047	-1.9393942	1.0364310
C	1.4079874	-0.4912738	-0.3726371
C	2.0785760	0.8714979	-0.5267933
O	2.3940230	1.1949086	-1.6660908
N	2.6681011	1.2909210	0.5838121
H	2.6471613	0.8137253	1.4611636
C	3.5992182	2.4272415	0.4978085
H	-1.2510190	-3.3635481	0.1120275
H	-2.7115584	-4.0643337	0.8759507
H	-1.8563051	-2.7040102	1.6507740
H	-4.7874839	-0.4771059	-1.0435005
H	-5.6763787	-0.7305469	0.4855418
H	-6.1021406	-1.6858639	-0.9697629
H	-0.3946941	1.7019424	2.5376432
H	-1.3013305	0.5535636	1.4968696
H	-2.1628934	1.7497527	2.5084033
H	-1.8155889	3.9357547	-1.4132762
H	-3.0772367	2.7579618	-1.8773844
H	-1.3852409	2.5129213	-2.4122435
H	4.7156363	-3.7100636	0.2341211
H	3.4137128	-3.5905935	1.4626630
H	4.6995420	-2.3521471	1.3848324
H	1.1375134	-0.7869791	-1.3921068
H	0.4805817	-0.4316889	0.2113429
H	3.2899057	3.0723096	-0.3285652
H	4.6249828	2.0828009	0.3000314
H	3.5752749	2.9943412	1.4358900

Table S2. Frame 2

0 1			
C	2.19639	-0.96100	3.03023
C	3.03639	-1.61100	1.93023
O	2.54638	-1.56100	0.80023
N	4.00638	-2.48100	2.22023
H	4.23639	-2.58100	3.19023
C	4.84638	-3.19100	1.25023
C	-0.07362	-2.60100	-0.70977
C	0.60638	-2.11100	-1.98977
O	0.12639	-2.32100	-3.09977
N	1.70638	-1.39100	-1.80977
H	2.03639	-1.35100	-0.86977
C	2.53639	-0.79100	-2.86977
C	-3.06362	3.14900	3.05023
C	-2.18362	3.02900	1.80023
O	-2.06362	3.95900	1.01023
N	-1.96361	1.75900	1.48023
H	-2.28362	1.06900	2.13023
C	-1.26362	1.28900	0.28023
C	-1.97361	1.45900	-1.06977
O	-1.53362	2.16900	-1.96977
N	-3.20362	0.95900	-0.98977
H	-3.47361	0.23900	-0.35977
C	-4.15362	1.12900	-2.08977
H	1.47961	-0.30071	2.58849
H	2.83486	-0.40701	3.68623
H	1.68689	-1.72044	3.58571
H	4.48994	-2.99682	0.26021
H	4.80471	-4.24239	1.44449
H	5.85738	-2.85183	1.33824
H	-0.94502	-3.16779	-0.96335
H	-0.35667	-1.75999	-0.11187
H	0.60529	-3.21789	-0.15893
H	2.05084	-0.91366	-3.81534
H	3.49016	-1.27542	-2.89309
H	2.67069	0.25164	-2.67038
H	-3.21997	4.18262	3.27845
H	-2.57823	2.67102	3.87536
H	-4.00670	2.67710	2.86913
H	-0.33584	1.81894	0.22280
H	-1.18040	0.23037	0.41170
H	-3.81584	1.91307	-2.73478
H	-5.11535	1.38164	-1.69462
H	-4.22429	0.21659	-2.64422

Table S3. Frame 3

0 1			
C	-1.1150537	-2.8470458	0.1274765
C	-2.4977627	-2.3051457	-0.2227522
O	-2.5678564	-1.1871958	-0.7547635
N	-3.5879852	-2.9954342	0.0756043
H	-3.4077870	-3.8481042	0.5557943
C	-4.9816989	-2.5213613	0.0507652
C	-2.0352304	1.4546593	1.8781358
C	-2.6137697	2.3377812	0.7743297
O	-2.5257752	3.5649425	0.8880496
N	-2.9594182	1.6219090	-0.2861202
H	-2.8550638	0.6127959	-0.2405206
C	-3.6245369	2.1081671	-1.5056981
C	5.2447245	-1.9851156	1.0336206
C	4.3013020	-1.3230042	0.0170761
O	4.7185965	-0.6016258	-0.8848542
N	3.0736144	-1.2315254	0.5127725
H	2.7553118	-1.8174923	1.2548143
C	2.0296465	-0.5525020	-0.2602466
C	2.3801573	0.8719601	-0.6875051
O	2.5381020	1.0631132	-1.8875942
N	2.4103662	1.7131915	0.3432311
H	2.2996002	1.3902719	1.2808098
C	2.7140286	3.1461605	0.2068898
H	-0.4787327	-2.7583766	-0.7595354
H	-1.1159670	-3.8875214	0.4808978
H	-0.7032549	-2.2038080	0.9156477
H	-5.0779665	-1.7638043	-0.7322670
H	-5.2634151	-2.0788213	1.0164561
H	-5.6401652	-3.3672830	-0.1731990
H	-2.1196146	1.9769390	2.8368176
H	-0.9666599	1.2981444	1.6685296
H	-2.5205511	0.4711703	1.9500837
H	-3.5743538	3.2017159	-1.5207358
H	-4.6773753	1.7918754	-1.5285853
H	-3.1109234	1.7080974	-2.3886540
H	6.1391876	-2.3686717	0.5293331
H	4.7742254	-2.7987546	1.6032910
H	5.5598640	-1.1988471	1.7323918
H	1.8267531	-1.0966390	-1.1905738
H	1.1160088	-0.5498545	0.3493481
H	2.7945570	3.3684802	-0.8613968
H	3.6659895	3.3915241	0.6976382
H	1.9089182	3.7487817	0.6459485

Table S4. Frame 4

0 1

C	2.34769	-0.44354	3.00923
C	3.29769	-1.11354	2.00923
O	2.90769	-1.15354	0.84923
N	4.33769	-1.73354	2.56923
H	4.52769	-1.69354	3.54923
C	5.19769	-2.65354	1.81923
C	0.28769	-2.51354	-0.55077
C	1.04769	-2.04354	-1.79077
O	0.46769	-2.42354	-2.81077
N	2.21769	-1.44354	-1.67077
H	2.54769	-1.42354	-0.73077
C	3.05769	-1.03354	-2.81077
C	-4.19231	2.88646	2.47923
C	-3.15231	2.82646	1.35923
O	-3.36231	3.54646	0.37923
N	-2.42231	1.71646	1.38923
H	-2.37231	1.11646	2.17923
C	-1.40231	1.45646	0.34923
C	-1.94231	1.26646	-1.07077
O	-1.61231	1.96646	-2.02077
N	-3.03231	0.50646	-1.08077
H	-3.42231	0.32646	-0.17077
C	-3.91231	0.17646	-2.21077
H	1.54631	0.02746	2.47927
H	2.88347	0.29165	3.57257
H	1.95040	-1.18220	3.67364
H	4.92569	-2.63260	0.78459
H	5.07592	-3.64605	2.20001
H	6.21926	-2.35356	1.92559
H	-0.63175	-2.97265	-0.84869
H	0.07948	-1.67421	0.07938
H	0.88329	-3.22278	-0.01492
H	2.50122	-1.13608	-3.71891
H	3.92817	-1.65428	-2.85384
H	3.35338	-0.01268	-2.68698
H	-4.74629	3.79871	2.40304
H	-3.69801	2.84748	3.42741
H	-4.86015	2.05517	2.39072
H	-0.72581	2.28537	0.33648
H	-0.94183	0.52792	0.61507
H	-3.41235	0.40129	-3.12968
H	-4.81098	0.75329	-2.14328
H	-4.15424	-0.86543	-2.18200

Table S5. Frame 5

0 1			
C	2.12323	-0.30831	2.82862
C	3.07323	-1.00831	1.84862
O	2.72323	-1.10831	0.67862
N	4.23323	-1.53831	2.24862
H	4.73323	-1.32831	3.08862
C	5.10323	-2.31831	1.35861
C	-0.17677	-2.96831	-0.43138
C	0.34323	-2.47831	-1.78139
O	-0.37677	-2.64831	-2.77138
N	1.52323	-1.87831	-1.80139
H	1.90323	-1.46831	-0.96139
C	2.13323	-1.44831	-3.06138
C	-3.18677	3.18169	2.81862
C	-2.31677	2.94169	1.57862
O	-2.26677	3.77169	0.67862
N	-1.83677	1.71169	1.48861
H	-2.02677	1.07169	2.22862
C	-0.99677	1.28169	0.35861
C	-1.76677	1.30169	-0.96139
O	-1.49677	2.16169	-1.80139
N	-2.90677	0.61169	-0.99138
H	-3.15677	0.25169	-0.09138
C	-3.86677	0.44169	-2.08138
H	1.25965	0.03956	2.30125
H	2.62457	0.52257	3.27939
H	1.82278	-0.99876	3.58882
H	4.57625	-2.54799	0.45615
H	5.38910	-3.22704	1.84584
H	5.97813	-1.74811	1.12550
H	-1.13312	-3.42961	-0.56366
H	-0.27058	-2.13827	0.23730
H	0.50911	-3.68030	-0.02208
H	1.65275	-1.94526	-3.87813
H	3.17430	-1.69539	-3.05624
H	2.01835	-0.39010	-3.17041
H	-3.53513	4.19339	2.81951
H	-2.60780	3.00305	3.70054
H	-4.02435	2.51608	2.80092
H	-0.15785	1.94120	0.28014
H	-0.68005	0.27650	0.54349
H	-3.46920	0.87135	-2.97706
H	-4.78427	0.93049	-1.82807
H	-4.04924	-0.60115	-2.23655

Table S6. Frame 6

0 1			
C	1.60331	-0.88854	3.09131
C	2.55331	-1.32854	1.98131
O	2.22331	-1.03854	0.83131
N	3.68331	-1.98854	2.25131
H	4.12331	-2.05854	3.14131
C	4.50331	-2.66854	1.24131
C	-0.20669	-2.57854	-1.02869
C	0.65331	-1.94854	-2.12869
O	0.15331	-1.83854	-3.24869
N	1.93331	-1.66854	-1.89869
H	2.23331	-1.79854	-0.94869
C	2.86331	-1.18854	-2.92869
C	-3.67669	2.50146	2.87131
C	-2.66669	2.55146	1.73131
O	-2.77669	3.34146	0.80131
N	-1.68669	1.65146	1.72131
H	-1.51669	1.19146	2.60131
C	-0.82669	1.31146	0.58131
C	-1.51669	1.54146	-0.76869
O	-1.10669	2.48146	-1.45869
N	-2.51669	0.74146	-1.11869
H	-2.77669	0.03146	-0.46869
C	-3.26669	0.68146	-2.37869
H	0.76540	-0.37952	2.66269
H	2.11689	-0.22978	3.76002
H	1.26144	-1.74776	3.62960
H	4.02824	-2.59188	0.28562
H	4.61062	-3.70008	1.50455
H	5.46875	-2.20930	1.19756
H	-1.20044	-2.72963	-1.39548
H	-0.23478	-1.92669	-0.18063
H	0.21442	-3.51936	-0.74160
H	2.35210	-1.11840	-3.86605
H	3.68050	-1.87340	-3.01848
H	3.23500	-0.22405	-2.65210
H	-4.39809	3.28124	2.74314
H	-3.16903	2.63495	3.80370
H	-4.17213	1.55308	2.86754
H	0.05378	1.91799	0.62364
H	-0.59279	0.26972	0.65181
H	-2.93910	1.46752	-3.02653
H	-4.31131	0.79687	-2.17781
H	-3.09650	-0.26375	-2.85040

Table S7. Frame 7

0 1

C	2.03546	-0.68992	2.99554
C	2.80546	-1.01992	1.71554
O	2.40546	-0.64992	0.60554
N	3.92546	-1.71992	1.88554
H	4.34546	-1.86992	2.77554
C	4.76546	-2.23992	0.79554
C	-0.19454	-2.85992	-1.08446
C	0.40546	-2.29992	-2.37446
O	-0.09454	-2.50992	-3.48446
N	1.62546	-1.77992	-2.27446
H	2.03546	-1.62992	-1.38446
C	2.27546	-1.19992	-3.46446
C	-3.20454	2.72008	3.45554
C	-2.36454	2.71008	2.17554
O	-2.35454	3.63008	1.35554
N	-1.80454	1.53008	1.93554
H	-1.96454	0.63008	2.34554
C	-0.98454	1.22008	0.75554
C	-1.63454	1.42008	-0.61446
O	-1.17454	2.17008	-1.48446
N	-2.76454	0.74008	-0.74446
H	-3.04454	0.13008	0.00554
C	-3.65454	0.61008	-1.90446
H	1.15798	-0.12927	2.74940
H	2.65717	-0.11170	3.64673
H	1.75267	-1.59798	3.48580
H	4.28316	-2.06125	-0.14274
H	4.91022	-3.29183	0.92756
H	5.71398	-1.74491	0.80898
H	-1.17104	-3.24882	-1.28474
H	-0.26392	-2.07986	-0.35536
H	0.43145	-3.64299	-0.71051
H	1.71125	-1.45258	-4.33781
H	3.26736	-1.59081	-3.55521
H	2.31936	-0.13558	-3.36375
H	-3.62865	3.69231	3.59624
H	-2.58289	2.47767	4.29201
H	-3.98913	1.99724	3.37292
H	-0.11515	1.84262	0.79417
H	-0.79086	0.16974	0.82024
H	-3.32819	1.27083	-2.68022
H	-4.65381	0.86301	-1.61745
H	-3.63038	-0.39838	-2.26130

Section S1.2. Coordinates for monomeric species:

Table S8. Frame 1

0 1			
C	-0.88250	-2.42794	2.40573
C	-1.36250	-1.68794	1.15573
O	-0.48250	-1.03794	0.59574
N	-2.63250	-1.29794	1.03574
H	-3.36250	-1.74794	1.53574
C	-3.17250	-0.45794	-0.04427
C	31.73750	1.79206	0.81573
C	31.92750	1.62206	-0.69426
O	32.53750	2.49206	-1.31426
N	31.21750	0.62206	-1.20427
H	30.51750	0.11206	-0.69426
C	31.13750	0.28206	-2.62426
H	0.15932	-2.65210	2.30949
H	-1.43448	-3.33798	2.51548
H	-1.03662	-1.81130	3.26649
H	-2.36634	0.01426	-0.56584
H	-3.81429	0.28991	0.37253
H	-3.72968	-1.06695	-0.72512
H	32.33443	2.61046	1.16042
H	32.03838	0.89548	1.31625
H	30.70694	1.98903	1.02560
H	30.49666	-0.56515	-2.75260
H	32.11527	0.04843	-2.99071
H	30.74203	1.11411	-3.16851

Table S9. Frame 2

0 1			
C	0.02000	-2.61471	2.48809
C	-0.56000	-2.34471	1.09809
O	0.14000	-1.66471	0.34809
N	-1.87000	-2.53471	0.97809
H	-2.39000	-3.25471	1.43809
C	-2.56000	-2.11471	-0.26191
C	0.30000	2.69529	0.82809
C	0.85000	2.60529	-0.59191
O	1.49000	3.50529	-1.12191
N	0.80000	1.39529	-1.14191
H	0.32000	0.63529	-0.69191
C	1.37000	1.08529	-2.46191
H	1.07439	-2.43301	2.47605
H	-0.16330	-3.63340	2.75933
H	-0.44551	-1.96652	3.20087
H	-1.99285	-1.34512	-0.74250
H	-3.53334	-1.74206	-0.01975
H	-2.65270	-2.95327	-0.92003
H	-0.22044	1.79142	1.06694
H	-0.37354	3.52377	0.89785
H	1.10819	2.83382	1.51550
H	1.20657	0.05214	-2.68727
H	2.42088	1.28646	-2.45279
H	0.89721	1.69176	-3.20593

Table S10. Frame 3

0 1			
C	-0.23338	-2.34103	2.23029
C	-0.95338	-1.62103	1.09029
O	-0.35338	-0.77103	0.44029
N	-2.22338	-1.97103	0.87029
H	-2.78338	-2.33103	1.61029
C	-3.03338	-1.44103	-0.22971
C	0.78662	2.23897	0.79030
C	1.40662	1.93897	-0.57970
O	2.49662	2.46897	-0.75970
N	0.90662	0.87897	-1.20970
H	0.19662	0.38897	-0.70970
C	1.13662	0.55897	-2.62971
H	0.76951	-1.97578	2.30579
H	-0.21611	-3.39262	2.03344
H	-0.74953	-2.15870	3.14967
H	-2.38990	-1.06689	-0.99837
H	-3.65333	-0.64824	0.13368
H	-3.64842	-2.22093	-0.62769
H	-0.15834	1.74344	0.87036
H	0.64580	3.29452	0.89463
H	1.43975	1.88829	1.56187
H	0.62131	-0.34485	-2.87964
H	2.18499	0.43091	-2.80119
H	0.77055	1.35851	-3.23933

Table S11. Frame 4

0 1			
C	-0.40147	-2.40971	2.49853
C	-0.94147	-1.71971	1.23853
O	-0.10147	-1.24971	0.47853
N	-2.26147	-1.54971	1.20853
H	-2.89147	-1.97971	1.84853
C	-2.87147	-0.81971	0.08853
C	1.41853	1.71029	0.80853
C	1.41853	1.83030	-0.72147
O	2.01853	2.71029	-1.34147
N	0.81853	0.81030	-1.33147
H	0.52853	0.02029	-0.81147
C	0.89853	0.65029	-2.79147
H	0.66240	-2.49953	2.42781
H	-0.83656	-3.38331	2.58626
H	-0.65376	-1.82735	3.35999
H	-2.10181	-0.40369	-0.52747
H	-3.48797	-0.03247	0.46944
H	-3.46880	-1.49141	-0.49191
H	1.91384	2.55855	1.23282
H	1.93197	0.81669	1.09620
H	0.40978	1.67197	1.16329
H	0.35522	-0.22359	-3.08480
H	1.92283	0.54820	-3.08349
H	0.47573	1.50988	-3.26817

Table S12. Frame 5

0 1			
C	-1.06044	-1.93735	2.63735
C	-1.50044	-1.32735	1.29735
O	-0.62044	-1.03735	0.48735
N	-2.72044	-0.79735	1.24735
H	-3.40044	-0.98735	1.94735
C	-3.14044	0.11265	0.17735
C	1.88956	1.40265	0.71735
C	2.01956	1.22265	-0.79265
O	2.82956	1.88265	-1.45265
N	1.23956	0.30265	-1.36265
H	0.72956	-0.30735	-0.76265
C	1.01956	0.19265	-2.81265
H	-0.06791	-2.32593	2.54356
H	-1.72921	-2.72825	2.90592
H	-1.07782	-1.18264	3.39564
H	-2.33550	0.24449	-0.51517
H	-3.40725	1.05938	0.59855
H	-3.98511	-0.30178	-0.33225
H	2.56508	2.16497	1.04514
H	2.12592	0.48161	1.20800
H	0.88654	1.68755	0.95754
H	1.63258	0.90685	-3.32160
H	-0.00975	0.38488	-3.03283
H	1.27579	-0.79381	-3.13845

Table S13. Frame 6

0 1			
C	-0.76824	-1.92809	2.70132
C	-1.36824	-1.17809	1.51132
O	-0.59824	-0.88809	0.59132
N	-2.66824	-0.89809	1.51132
H	-3.24824	-1.36809	2.17132
C	-3.29823	-0.09809	0.45132
C	2.01176	1.66191	0.47132
C	1.99176	1.19191	-0.98868
O	2.89177	1.57191	-1.72868
N	0.98176	0.47191	-1.46868
H	0.30176	0.07191	-0.85868
C	0.83176	0.15191	-2.89868
H	0.28012	-2.07044	2.54136
H	-1.24638	-2.88014	2.80072
H	-0.91863	-1.35845	3.59452
H	-2.53996	0.33568	-0.16655
H	-3.88635	0.67913	0.89286
H	-3.92700	-0.72670	-0.14398
H	2.90485	2.22241	0.65337
H	1.98591	0.81148	1.12017
H	1.15849	2.27970	0.65886
H	1.61027	0.63090	-3.45492
H	-0.11931	0.49976	-3.24415
H	0.89538	-0.90737	-3.03571

Table S14. Frame 7

0 1			
C	-0.70809	-1.97618	2.81971
C	-1.33809	-1.37618	1.55970
O	-0.58809	-1.10618	0.61971
N	-2.61809	-1.02618	1.57970
H	-3.13809	-1.19618	2.41970
C	-3.31809	-0.22618	0.56971
C	1.97191	1.52382	0.38971
C	2.05191	1.28382	-1.12029
O	2.92191	1.82382	-1.79029
N	0.95191	0.67382	-1.55029
H	0.26191	0.42382	-0.87029
C	0.65191	0.35382	-2.95029
H	0.32129	-2.19982	2.63194
H	-1.22575	-2.87449	3.08420
H	-0.77956	-1.27350	3.62347
H	-2.68691	-0.09912	-0.28491
H	-3.56107	0.73201	0.97926
H	-4.21708	-0.72765	0.27775
H	2.86319	2.01483	0.72052
H	1.87279	0.58596	0.89516
H	1.12385	2.13841	0.60873
H	1.46423	0.67061	-3.57053
H	-0.24260	0.85989	-3.24805
H	0.51530	-0.70240	-3.05336

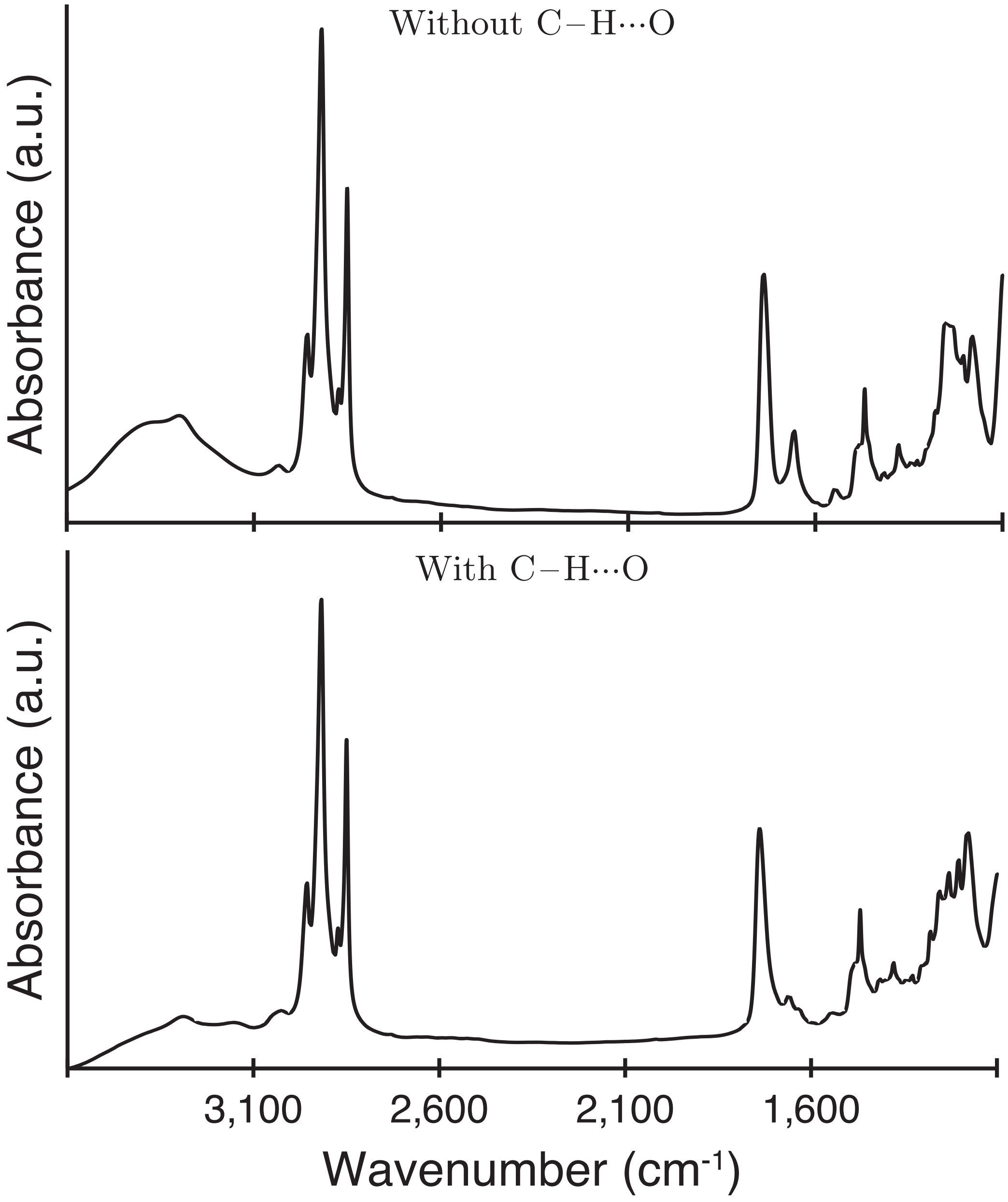

Figure S1. ATR-IR spectra of the glycophorin A transmembrane domain edited with the $^{13}C=^{18}O$ label at Val80 in hydrated lipid bilayers. Upper panel: Full spectrum of the glycophorin A monomeric peptide with the G79L mutation. Lower panel: Full spectrum of the C−H···O dimeric hydrogen bonded species.

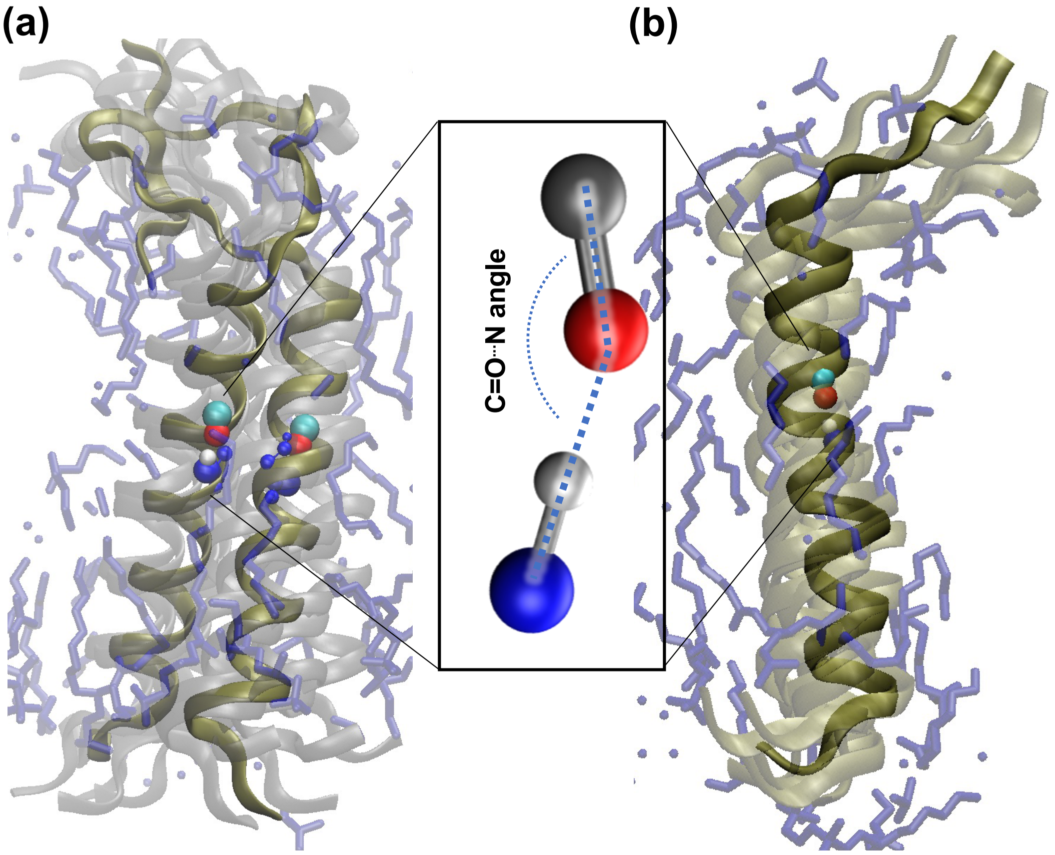

Figure S2. MD-simulated structures of (a) glycophorin-A dimer and (b) monomer containing G79I mutation. The moieties in ball and stick models within the helices represents Val80 C=O and Val84 N−H with C, O , H and N atoms marked as cyan, red, white and blue respectively. The inset picture shows the canonical *i* to *i*-4 H-bond between Val80 C=O and Val84 N−H, with C, O, H, and N atoms as grey, red, white and blue respectively. C-O-N angle has been marked and defined as the antecedent angle within the text.

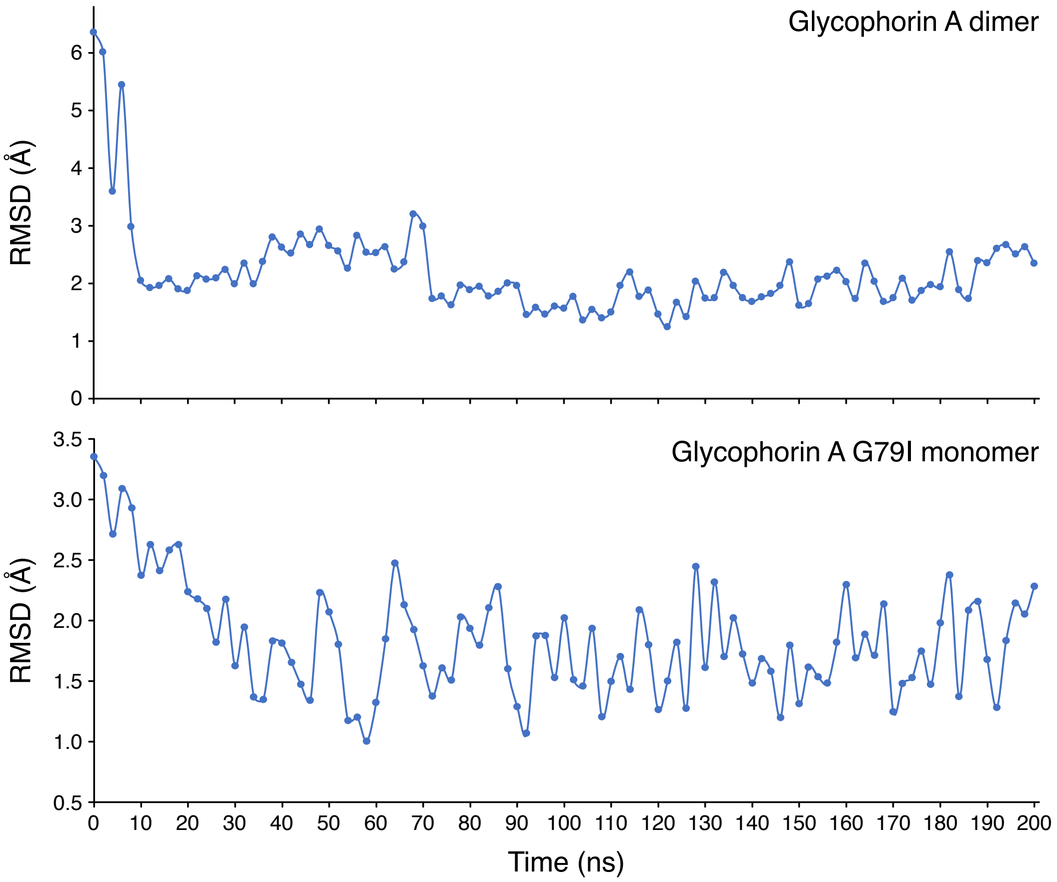

Figure S3. Root mean square deviation (Å) relative to the experimentally determined structure versus time (ns) plot for the molecular dynamic (MD) simulation of glycophorin-A (dimeric) and glycophorin-A G79I (monomeric) proteins within DMPC bilayer for 200 ns.

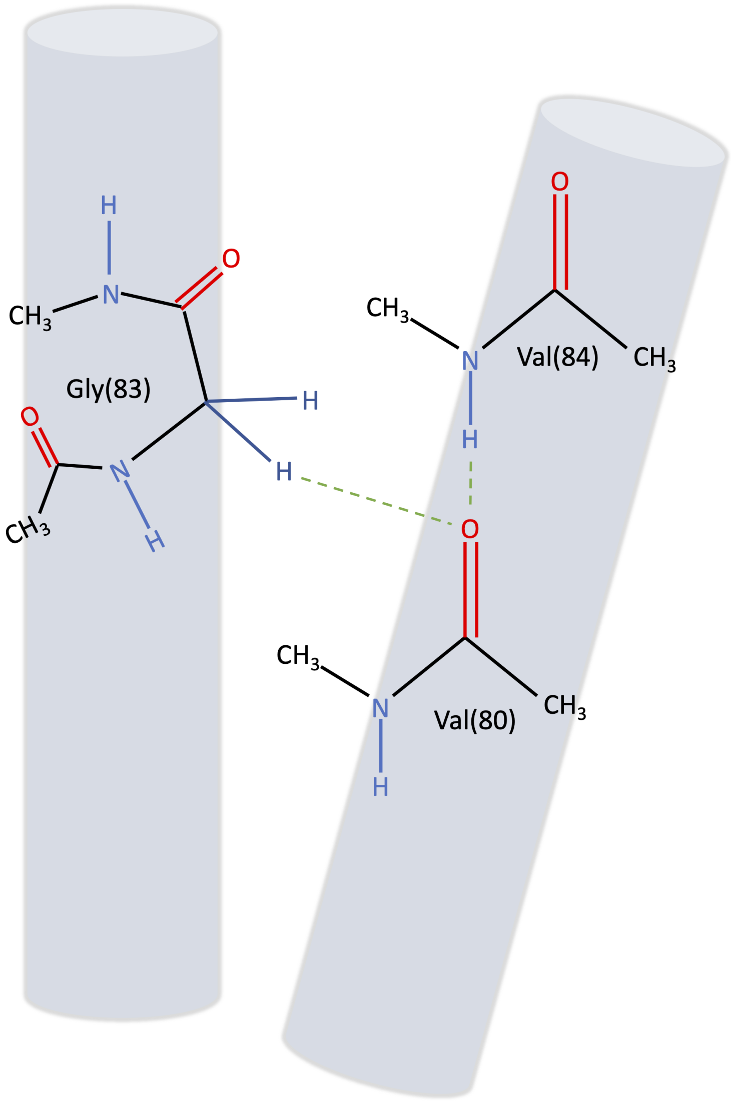

Figure S4. Chemical structures of the two N-methylacetamides representing Val80 and Val84 of chain A, together forming the canonical α-helical backbone H-bond, and acetylglycinemethylamide representing Gly79 from chain B that serves as the H-bond donor of the noncanonical inter-helical Cα-H···O=C hydrogen bond. H-bonds are shown as green dashed lines.

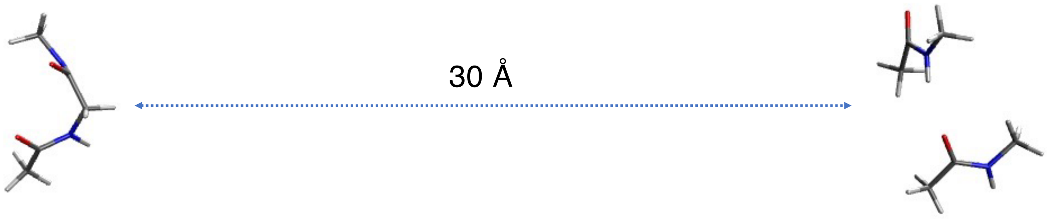

Figure S5. Model showing how the H-bond strength was measured upon separation of the C–H···O H-bond.
